# A Role for Nrf2 in Redox Signalling of the Invasive Extravillous Trophoblast in Severe Early Onset IUGR Associated with Preeclampsia

**DOI:** 10.1371/journal.pone.0047055

**Published:** 2012-10-09

**Authors:** Nisreen Kweider, Berthold Huppertz, Christoph Jan Wruck, Rainer Beckmann, Werner Rath, Thomas Pufe, Mamed Kadyrov

**Affiliations:** 1 Department of Anatomy and Cell Biology, Medical Faculty, RWTH Aachen University, Aachen, Germany; 2 Institute of Cell Biology, Histology and Embryology, Medical University of Graz, Graz, Austria; 3 Department of Obstetrics and Gynecology, University Hospital of the RWTH, Aachen, Germany; 4 MEDIAN Kliniken, Baden-Wuerttemberg, Germany; VU University Medical Center, The Netherlands

## Abstract

**Background:**

Preeclampsia (PE) is characterized by increased lipid oxidation and diminished antioxidant capacity, while intrauterine growth restriction (IUGR) is characterized by impaired invasion of the extravillous trophoblast. Vascular endothelial growth factor (VEGF) has been reported to be altered in preeclampsia. A relationship between VEGF and nuclear factor erythroid 2-related factor-2 (Nrf2) has been shown in vitro, where VEGF prevents oxidative damage via activation of the Nrf2 pathway. In this study the expression of Nrf2, VEGF and 4-hydroxynonenal (4-HNE), was determined in interstitial and endovascular/intramural extravillous trophoblast (EVT) in normal pregnancies and those complicated by severe early onset IUGR associated with preeclampsia IUGR/PE.

**Materials and Methods:**

Full-thickness uterine tissues derived from caesarean hysterectomies performed in 5 healthy normotensive women delivering term infants and 6 women with severe early onset IUGR with preeclampsia (29–34 weeks gestation) were analyzed. Interstitial and endovascular extravillous trophoblast were quantified after immunohistochemical staining of paraffin sections using antibodies against Nrf2, 4-HNE, VEGF, and cytokeratin 7.

**Results:**

Uterine tissues from women suffering from severe early onset IUGR/PE were characterized by reduced invasion of extravillous trophoblast into the endometrial and myometrial segments of spiral arteries in the placental bed. Extravillous trophoblast showed an increased cytoplasmic expression of Nrf2 and 4-HNE in IUGR/PE cases. The increased expression of Nrf2 in cases of IUGR/PE was associated with decreased expression of VEGF in these cells compared to controls.

**Conclusion:**

Our data suggests that besides villous cytotrophoblast, also the extravillous trophoblast is a source of Nrf2-dependent genes. VEGF deficiency may cause higher oxidative stress in extravillous trophoblast in cases with IUGR/PE. The resulting reduced basal defence against oxidative stress and the higher vulnerability to oxidative damage may play a role in the limited trophoblast invasion into spiral arteries in cases suffering from severe early onset IUGR/PE.

## Introduction

The placenta is the key organ for successful pregnancy and fetal growth. It performs key transport, metabolic, and secretory functions to support fetal development. Term placental villi are covered by the multinucleated syncytiotrophoblast that shares a basement membrane with a subjacent, discontinuous layer of cytotrophoblast. The syncytiotrophoblast is in direct contact with maternal blood and regulates maternal-fetal exchange [1].

To allow the efficient supply with oxygen and other key molecules to the placenta and hence the fetus, the invasive cell population of the extravillous trophoblast (EVT) invades the uterine decidua and myometrium (interstitial trophoblast). A subgroup of the interstitial trophoblast migrates towards the uterine spiral arteries, reaches the walls of such arteries (intramural trophoblast) and can be found in the lumen of such vessels (endovascular trophoblast), transforming them into large conduit vessels of low resistance [2].

**Figure 1:**
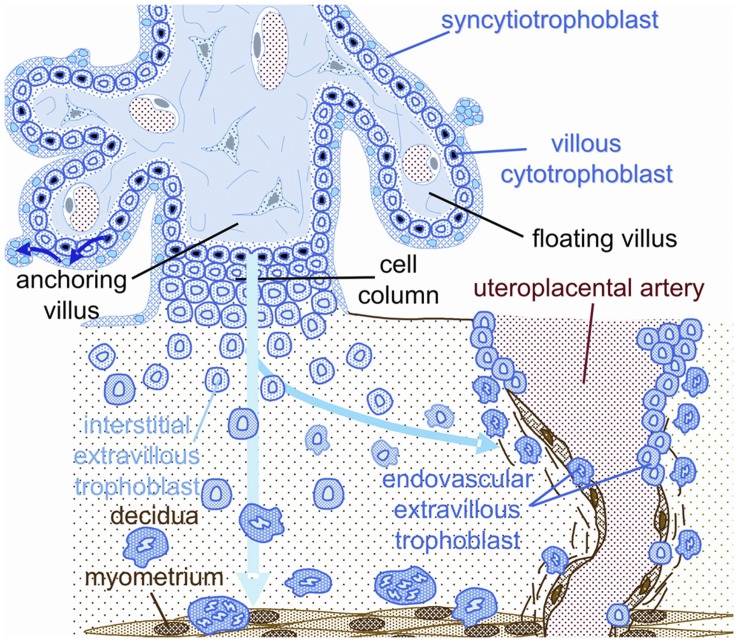
Schematic representation of the invasion route of interstitial and endovascular trophoblast in human pregnancy. Blue: fetal tissues, including interstitial trophoblast and its intravasating derivatives. Brown: maternal tissues. Endovascular trophoblast is derived from a side route of interstitial trophoblast (Kaufmann et al. 2003, modified).

This physiological transformation is characterized by a gradual loss of the normal musculo-elastic structure of the arterial wall and replacement by amorphous fibrinoid material in which trophoblast cells are embedded [3], [4], [5], [6], [7]. The interstitial route of trophoblast invasion through the placental bed has been well described by Kaufmann et al. figure (1) [5]. These physiological changes are thought to be required for a successful pregnancy.

At the same time, proliferation, migration, and invasion of extravillous trophoblast are regulated by a large number of locally derived molecules including members of the VEGF and the angiopoietin families to maintain a healthy uteroplacental homeostasis [4], [8]. Extravillous trophoblast dysfunction has been implicated in IUGR, one of the leading syndromes causing preterm delivery and perinatal morbidity [9]. This dysfunction is characterized by reduced numbers of both, interstitial and endovascular trophoblast [10], [11], [12]. Severe early onset IUGR is often associated with preeclampsia, a leading cause of maternal death worldwide. In preeclampsia, hypertension is associated with widespread maternal endothelial dysfunction, leading to significant maternal morbidity [13].

Oxidative stress of the placenta is considered to be a key intermediate step in the pathogenesis of preeclampsia, but the cause for this stress remains unknown. In about 80% of all preeclampsia cases the extravillous trophoblast is not affected, while the other 20% of preeclampsia cases also suffer from IUGR with trophoblast malinvasion [14]. In any case, placental hypoxia does not occur in cases with trophoblast malfunction such as IUGR and preeclampsia [7], [14]. Hence, the cause for the presence of oxidative stress with increased lipid peroxidation products [15], [16], [17] in preeclampsia remains unknown. It is also still unknown whether in cases with IUGR and preeclampsia, the malfunctional extravillous trophoblast also suffers from oxidative stress.

A sensitive marker of oxidative damage and lipid peroxidation is 4-hydroxy-2-nonenal (4-HNE), a highly toxic aldehyde product of lipid peroxidation which can be evaluated by immunohistochemical staining [18], [19]. Significantly higher levels of 4-HNE have been consistently reported in pathological placentas associated with oxidative stress [20], [21].

A battery of genes encoding antioxidant enzymes is orchestrated upon exposure to reactive oxygen species (ROS). This coordinated response is regulated via the antioxidant response element (ARE) contained within the regulatory regions of so-called “safeguard” genes such as glutathione peroxidase, and heme oxygenase-1 (HO-1) [22]. Activation of the nuclear factor erythroid 2-related factor-2 (Nrf2) as a consequence of oxidative stress initiates and enhances transcription of these safeguard genes, thus protecting cells against oxidative stress as well as a wide range of other toxins [22], [23], [24].

Mann et al. were the first to discuss a link between Nrf2, vascular homeostasis, and preeclampsia [25]. Our laboratory provided the first experimental data that Nrf2 is active exclusively within villous cytotrophoblast of the preeclamptic placenta [26], strongly suggesting that these cells suffer from oxidative stress. Loset et al. [27] reported that the Nrf2-mediated oxidative stress response was overrepresented in the decidua of patients with preeclampsia, without indicating the presence or absence of IUGR.

Furthermore, in the human placenta VEGF, PlGF and their two receptors are differentially expressed throughout gestation. VEGF expression in the placenta and placental bed declines as pregnancy advances [28], [29], while PlGF and Flt-1 increase towards term [30]. Moreover, a recent study has shown that VEGF prevents oxidative damage via activation of the Nrf2 pathway in the choriocarcinoma cell line BeWo [31]. This suggests that that decreased VEGF bioavailability during preeclampsia could result in reduced basal defense against oxidative stress.

As an in vitro interplay was established between Nrf2 and VEGF [31], we hypothesized that severe early onset IUGR and preeclampsia could be associated with alterations in Nrf2 expression in the placental bed, particularly in the extravillous trophoblast, since it is well known that VEGF and its receptor VEGFR-1 (Flt-1) are expressed in these cells [32].

To test this hypothesis we used immunohistochemistry to examine the expression of Nrf2, VEGF, and the oxidative stress marker 4-HNE in third trimester placental bed tissues in cases of severe early onset IUGR/PE and control pregnancies.

## Materials and Methods

### Tissues

All samples were collected from caesarean hysterectomy subjects received for pathological examination at the Medical Institute, Ashgabat, Turkmenia (by M.K.) apart from 2 control subjects collected in Aachen, Germany. These materials were used in a former study by Kadyrov et al. [33]. In all instances, permission was granted for the histological studies, regarding the samples that were collected in Turkmenia, approval was obtained from the Ethics Committee of the mother and child medical centre in Ashgabat; protocol Nr. 047/1991 and Nr. 101/1992. For the other samples our protocol was approved by the Ethics Committee of the Medical Faculty of the University of Technology, Aachen, Germany (EK 512). Written informed consent was obtained from each patient enrolled in this study.

Uterine tissues of control subjects were derived from caesarean hysterectomies performed in five healthy normotensive women delivering term infants (38–40 weeks gestation) for reasons unrelated to placental development (fibroids, cervical carcinoma, or uterine atony after caesarean section).

Uterine tissues of pathological subjects were collected from six women with severe early onset IUGR and preeclampsia (29–34 weeks gestation). The selected criterion for complicated pregnancies was severe preeclampsia with IUGR. Severe Preeclampsia was defined following the criteria of the American College of Obstetricians and Gynecologists [34], new onset hypertension (systolic blood pressure ≥160 mmHg or diastolic blood pressure ≥110 mmHg at least twice, measured six hours apart) and proteinuria (5 g or higher per 24-hour period) after 20 weeks of gestation. IUGR was defined as birth weight below the 10th centile [35] of customized birth weight for gestational age. The clinical characteristics of patients enrolled in this study are summarized in Table 1.

**Table 1:** Clinical characteristics of control and pathological pregnancies.

Clinical features	Normal pregnancy (n = 5)	Preeclampsia with IUGR (n = 6)
Maternal age (years)	33±4,570	32,5±2,876
Gestational age (wk)	38,2±1,3	31,5±2,428
Birth weight	3370±233,452	1908,33±270,955#
Systolic blood pressure (mm Hg)	112±8,366	160,5±11,726***
Diastolic blood pressure (mm Hg)	66±5,477	111,833±8,975#
Proteinuria number and percent %		
3–5 g/24 h	0 (0)	2 (33,33)
>5 g/24 h	0 (0)	4 (66,66)

Values were presented as mean ± SD or n (%).

*** P<0.005 when compared with normal pregnancy.

# P<0.001 when compared with normal pregnancy.

### Immunohistochemistry

The placental bed was identified macroscopically and later verified immunohistochemically by identification of extravillous trophoblast expressing cytokeratin 7.

**Figure 2:**
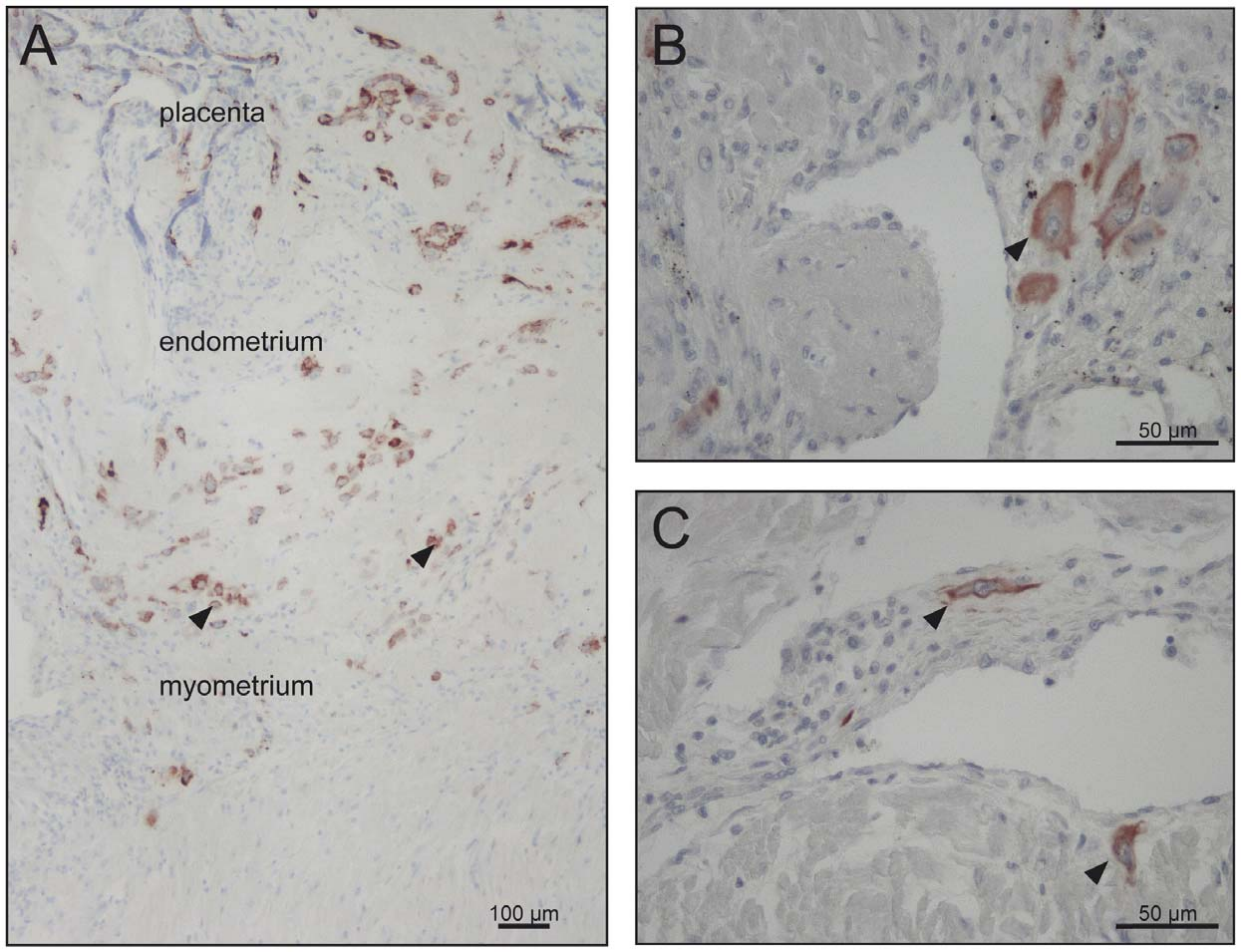
Invasion of extravillous trophoblast. Immunohistochemistry using an antibody directed against cytokeratin 7 revealed the invasive depth and the numerical density of invasive cells in control cases (A, B) and early onset IUGR/PE (C). Original magnification 400× (B, C). (A) In order to allow publication of a survey picture illustrating the complete invasive pathway, immunoreactivity of cytokeratin 7-positive cells was enhanced by image analysis. Figure A represents the placental, endometrial and myometrial parts of the sample Original magnification 100×.

At least five samples measuring about 2×2×2 cm per uterus, covering the full thickness of the uterine wall of the placental bed, were dissected, and fixed in 4% neutrally buffered formalin. The tissues were oriented during embedding in such a way that the uterine layers were perpendicular to the plane of section. To prevent bias each tissue block was rotated randomly around a virtual axis from the endometrium to the perimetrium and embedded vertically in paraffin at 56°C. All the uterine layers can be seen in figure (2 A) revealing the distribution of extravillous trophoblast (positively stained) that invaded perpendicularly from the endometrial-myometrial border into the myometrium.

Routine immunohistochemical procedures were performed on serial sections of 5 µm thickness with antibodies against cytokeratin 7 (trophoblast marker; clone OV_TL 12/30, dilution 1∶200, DAKO, Denmark), VEGF (sc- 7269, 1∶30, Santa Cruz, USA), Nrf2 (ab31163, 1∶50, Abcam, UK), and 4-HNE (ab46545, 1∶200, Abcam, UK). Binding of species-specific biotinylated secondary antibodies was visualised with AEC substrate chromogen (AEC) (Invitrogen, Germany). Sections were counterstained with hematoxylin.

### Evaluation of Immunohistochemical Staining

Each immunostained section was analyzed semi-quantitatively using a modification of the “quick score” method described by Detre et al. [36]. In brief: An intensity score was made on the basis of the average intensity of staining: 0 =  negative, 1 =  weak, 2 =  intermediate and 3 =  strong, then the percentage of positive cells (endovascular and interstitial, mononuclear and multinuclear extravillous trophoblast cells) for each staining was rated as: 1 = 0–25%, 2≤25–50%, 3≤50–75%, and 4≤75–100%. The whole of the section was assessed.

Two independent pathologists examined the immunohistochemical slides while blinded to the clinical history of the patients. The intensity score and the proportion score were then multiplied and scores summed to give a range of the possible score of 0 to 15. For example, negative staining in 25% of the extravillous trophoblast (0×1 = 0), weak staining in 50% (1×2 = 2) and strong staining in 25%(3×1 = 3) would give a total score of 0+2+3 = 5.

### Statistical Analysis

Statistical analyses were performed using Student’s unpaired t test for dual comparisons. Mean differences were considered to be significant when p<0.05. All statistical graphs and analyses were created with GraphPad Prism 5.0 (GraphPad Software, La Jolla, CA).

## Results

**Figure 3:**
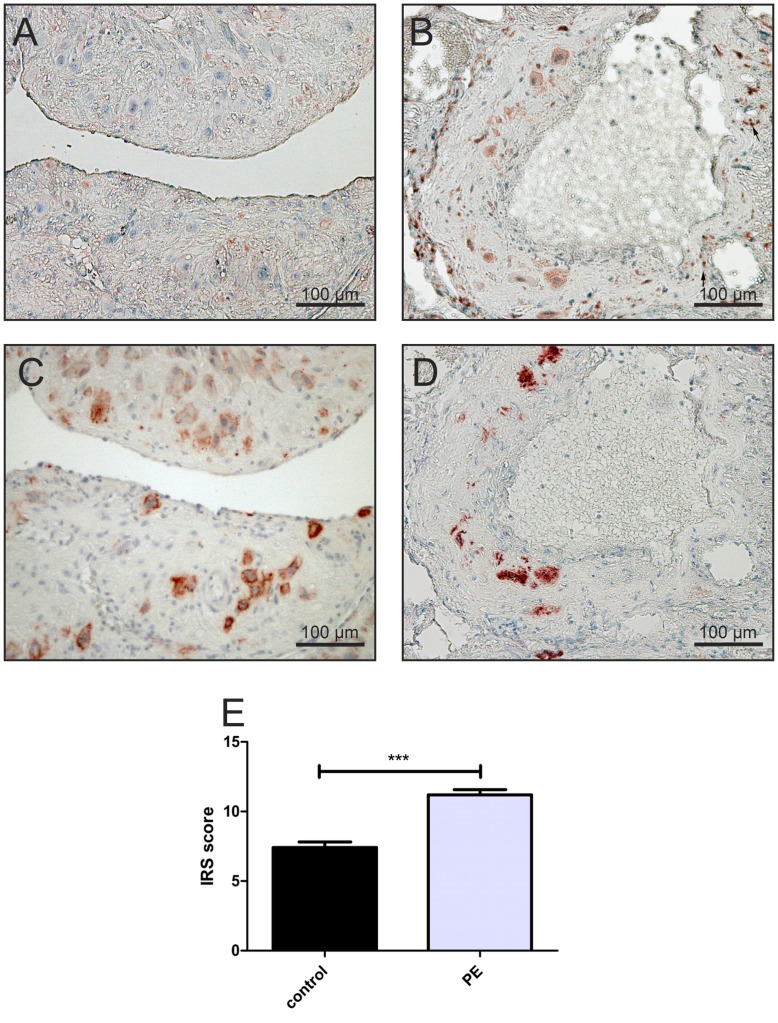
Expression of Nrf2 and cytokeratin 7 in the placental beds from patients with early onset IUGR/PE and controls with Immunoreactive Score (IRS) of staining for Nrf2. In placental beds of IUGR/PE cases, cytokeratin 7-positive cells (D) surround a spiral artery, reveals a strong cytoplasmic immunopositivity for Nrf2 (B). In contrast, very weak immunostaining is seen (A) in the same cytokeratin 7-positive cells (C) in control placental beds. Blue haematoxylin counterstain was performed. Original magnification 200×(bar = 100 µm). Mean score + (SEM) of Nrf2 immunolocalisation to endovascular and interstitial trophoblast cells in both control and early onset IUGR/PE groups (E) confirms the previously results. ***p<0.0001 versus the control group using Student’s t test. The arrows in (B) represent the leukocyte populations in decidua and myometrium, which were also positive for Nrf2 but they were excluded from the scoring.

**Figure 4:**
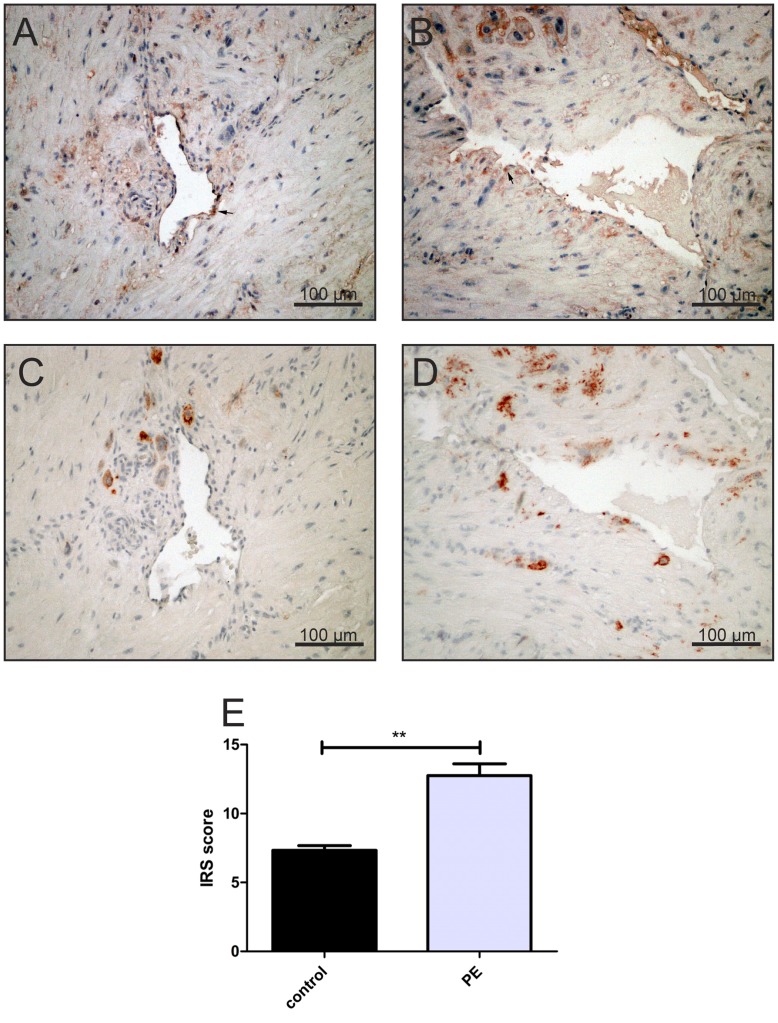
Comparison of 4-HNE expression in the placental beds between the control and IUGR/PE groups. Parallel sections of placental beds of normal pregnancy (C) and IUGR/PE (D) stained with cytokeratin 7 antibody, 4-HNE antibody (A, B) revealed that 4-HNE immunopositivity (red staining) is strongly expressed in extravillous trophoblast in IUGR/PE (B), and only very weak in healthy placental beds (A), Original magnification 200×, bar represents 100 µm. Other cells such as endothelial cells showed strong immunopositivity for 4-HNE (A, B arrows) but were dropped from the scoring. The IRS score showed a significant increase in the 4-HNE expression in the extra villous trophoblast in IUGR/PE cases when compared with the control groups. **p<0.005 when compared to the controls.

**Figure 5:**
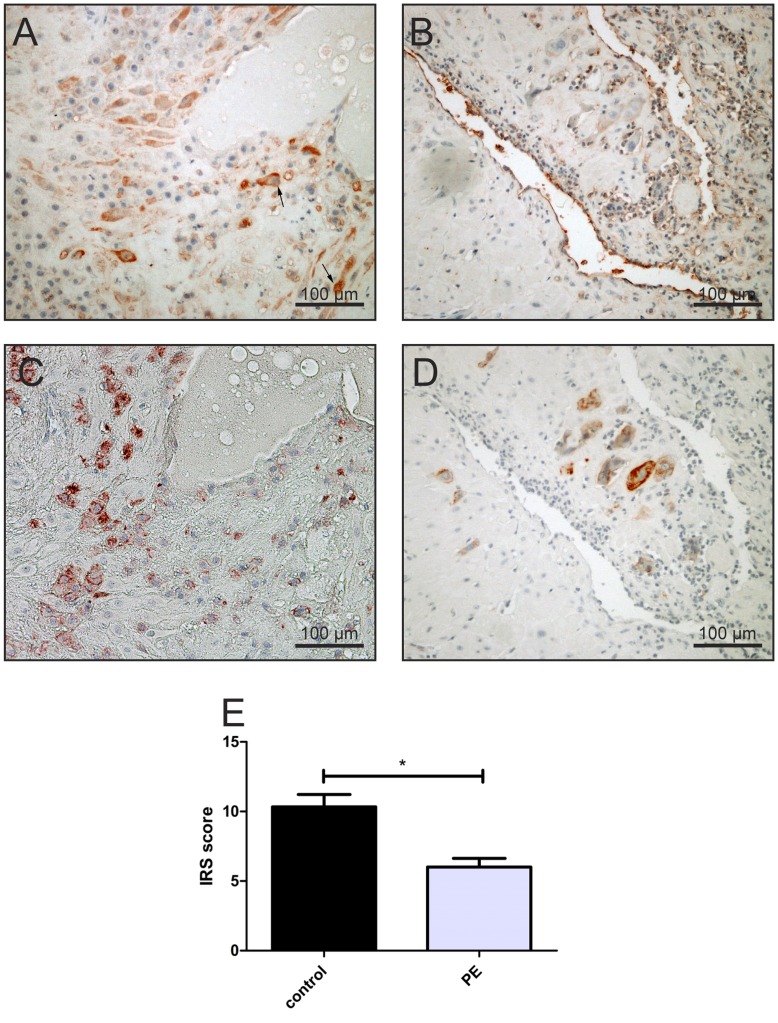
Expression of VEGF in the placental beds of women suffering from IUGR/PE or controls and the Immunoreactive Score (IRS) of this staining in the EVT. Interstitial trophoblast in controls display a stronger immunopositivity for VEGF (A), which are positive for cytokeratin 7 (C) than in early onset IUGR/PE samples (B). In control pregnancies decidual cells showed also a higher VEGF activity (A, arrows) but these cells were not analyzed. Original magnification 200×(bar = 100 µm). (E) The figure represent the mean score + (SEM) of VEGF immunolocalisation to endovascular and interstitial trophoblast cells in both control and early onset IUGR/PE groups. *p<0.05 between the two groups using Student’s t test.

Several different cell types in the placental beds and uterine wall biopsies were positive for Nrf2, 4-HNE and VEGF, including extravillous trophoblast figure (3 B, 4 B, 5 A), decidual stromal cells figure (5 A arrows), myometrial cells, leukocytes in decidua and myometrium figure (3 B arrows), and vascular endothelial cells figure 4 (A, B arrows). Since the aim of this study was to evaluate the differential immunostaining of the aforementioned proteins in extravillous trophoblast, other cells were not analyzed in detail. Semi quantitative analysis was confined to endovascular/intramural and interstitial extravillous trophoblast.

In control pregnancies, extravillous trophoblast expressed Nrf2 to a similar extend as has been described previously for villous trophoblast by Wruck et al. [26]. However, immunostaining for Nrf2 was stronger in endovascular and interstitial trophoblast in pregnancies complicated by early onset IUGR/PE figure (3 B). Immunostaining scores showed that immunostaining of cytoplasmic Nrf2 in extravillous trophoblast was significantly increased in the IUGR/PE group (p<0.0001) when compared with the control group figure (3 E).

In control pregnancies, interstitial and endovascular trophoblast, which stained positive for cytokeratin 7 figure (4 C), were virtually negative for 4-HNE figure (4 A). By contrast, these cytokeratin 7-positive cells figure (4 D) showed stronger 4-HNE immunoreactivity in IUGR/PE cases figure (4 B) in comparison with the same cell type of the control group. Immunostaining scores showed that immunostaining of 4-HNE in extravillous trophoblast was significantly increased in the IUGR/PE group (p<0.005) when compared with the control group figure (4 E).

VEGF was immunolocalized in both populations of the extravillous trophoblast, interstitial and endovascular trophoblast of the placental bed of the control group figure (5 A). VEGF immunostaining was almost absent on extravillous trophoblast from IUGR/PE cases figure (5 B). Semi quantitative analysis of VEGF immunostaining revealed a statistically significant difference between the control and IUGR/PE groups (p<0.05) figure (5 E).

## Discussion

The feto-placental circulation in severe early onset IUGR/PE cases is characterized by abnormal umbilical blood flow velocity waveforms, thought to be indicative of increased placental resistance [37]. In such cases, the uteroplacental blood is altered as well showing a ten-fold increased blood flow velocity from the spiral arteries into the intervillous space [7]. The distorted blood flow through spiral arteries that have not been transformed adequately may also induce changes in oxygen delivery to the surrounding decidual tissues. This in turn may result in a marked overproduction of ROS (reactive oxygen species), generated mainly in mitochondria, and will cause oxidative stress [38]. Increased production of ROS will trigger a cascade of events to enhance the cellular defense against oxidative stress, mainly by the Nrf2/ARE system. This is the first study to examine the expression of Nrf2 in the placental bed of IUGR/PE samples. As the transformation of spiral arteries is mediated by invasive extravillous trophoblast, we focused on the expression of Nrf2 in these cells.

Although our data were limited because of the shorter duration of gestation in the early onset IUGR/PE group, it is very difficult to obtain a normal control group of the same gestational age. At the same time, a comparison between specimens from early onset IUGR/PE and normal term controls does not necessarily limit the significance of the data because transformation of the myometrial spiral arteries largely occurs during the transition between the first and second trimester [39]. Consequently, uterine artery Doppler waveforms can identify a high proportion of women who develop early onset IUGR/PE already at 12 weeks [40], while they show low resistance patterns in normal pregnancies at 22–24 weeks [41]. Therefore, trophoblast populations and spiral artery modifications are very unlikely to change further between 30 and 40 weeks of gestation.

Endovascular and interstitial extravillous trophoblast populations show decreased densities in early onset IUGR/PE as already described by Kadyrov et al. [11]. In these pathological samples the extravillous trophoblast revealed a significant increase in staining intensity for Nrf2 in the cytoplasm of these cells figure 3 (B, E). This data suggests that the extravillous trophoblast in IUGR/PE suffers from (oxidative) stress leading to increased Nrf2 expression.

Activation of Nrf2 has been correlated with transfer of this protein into the nucleus [42] which does not become obvious in our study. It seems as if Nrf2 is not activated in these cells similar to what has been described for Nrf2 in preeclamptic villous trophoblast [43] These authors described lower transfer of Nrf2 into the nucleus, indicative for a lower activity of Nrf2. Hence, although the extravillous trophoblast in IUGR/PE upregulates Nrf2 expression it seems as if activation of Nrf2 fails under these conditions and thus a defense system to combat oxidative stress in the extravillous trophoblast is not effective.

Vascular endothelial growth factor (VEGF)-A is expressed by extravillous trophoblast and binds to the fms-like (Flt1) and kinase-insert domain-containing tyrosine kinase receptors, which are expressed on extravillous and villous trophoblast [32], [44]. In cases with early onset IUGR/PE, which are associated with a failure in spiral artery invasion [11], there is down-regulation of VEGF in the extravillous trophoblast and placental up-regulation of soluble Flt1 [45], [46]. In an earlier study we found that VEGF induces Nrf2 activation leading to prevention of oxidative stress [31]. Therefore, we tested whether increased Nrf2 expression is associated with higher immunostaining for VEGF in these cells.

In contrast to Nrf2, VEGF expression was reduced in extravillous trophoblast in the placental bed of IUGR/PE pregnancies figure (5 B) confirming already published data [45], [46]. This data suggests that Nrf2 activation is not a consequence of increased VEGF expression, but may be a secondary adaptive response to ROS signaling.

Since Nrf2 upregulates the expression of antioxidative and detoxifying enzymes, we further tested the level of oxidation-mediated changes in lipids (4-HNE) in extravillous trophoblast in term controls and IUGR/PE by immunohistochemistry figure 4 (A, B). There was a marked increase in 4-HNE immunostaining in extravillous trophoblast of IUGE/PE compared with the control group figure (4 B).

The picture that develops from our data is as follows: In IUGR/PE extravillous trophoblast experience oxidative stress (increased 4-HNE) and try to counteract by increased expression of Nrf2. However, since these cells fail to upreguate VEGF at the same time, activation of Nrf2 does not occur. In addition, several studies have shown that IUGR/PE is associated with reduced levels of antioxidant enzymes, which lead to further trophoblast damage [47], [48]. This in turn may result in increased apoptosis of extravillous trophoblast and decreased densities of such cells in the placental bed [11].

Taken together, we suggest that the extravillous trophoblast at late stages of IUGR/PE pregnancies demonstrate an impairment of the Nrf2 signalling pathway, in spite of the increased cytoplasmic Nrf2 expression, related to the cellular oxidative damage occurring at earlier stages of the syndrome.

In conclusion, it can be hypothesized that decreased VEGF bioavailability during early stages of preeclampsia results in insufficient Nrf2 activation, reduced basal defense against oxidative stress and a higher vulnerability of trophoblast to oxidative cell damage. This does not seem to be true for the villous trophoblast only but may be extended to the extravillous trophoblast in cases which combine IUGR and preeclampsia. The resulting damage causes increased apoptosis and will further speed the vicious circle of shallow invasion in such cases. Consequently, one would expect that these disturbances will limit trophoblast invasion into the walls of spiral arteries of women destined to develop early onset IUGR/PE. Specific attempts to strengthen the fetal endogenous defence system against oxidative stress during early gestation could prove to be a possible treatment option and may in turn reduce the risk of the combination of IUGR and preeclampsia and associated perinatal complications.
